# Role and Potential Mechanisms of Nicotinamide Mononucleotide in Aging

**DOI:** 10.14336/AD.2023.0519-1

**Published:** 2024-04-01

**Authors:** Sajid Ur Rahman, Abdul Qadeer, Ziyun Wu

**Affiliations:** ^1^Department of Food Science and Engineering, School of Agriculture and Biology, Shanghai Jiao Tong University, Shanghai 200240, China.; ^2^Institute for Infectious Diseases and Vaccine Development, Tongji University School of Medicine, Shanghai, 200072, China.

**Keywords:** aging, age-related diseases, nicotinamide mononucleotide, mechanisms, lifespan extension

## Abstract

Nicotinamide adenine dinucleotide (NAD^+^) has recently attracted much attention due to its role in aging and lifespan extension. NAD^+^ directly and indirectly affects many cellular processes, including metabolic pathways, DNA repair, and immune cell activities. These mechanisms are critical for maintaining cellular homeostasis. However, the decline in NAD^+^ levels with aging impairs tissue function, which has been associated with several age-related diseases. In fact, the aging population has been steadily increasing worldwide, and it is important to restore NAD^+^ levels and reverse or delay these age-related disorders. Therefore, there is an increasing demand for healthy products that can mitigate aging, extend lifespan, and halt age-related consequences. In this case, several studies in humans and animals have targeted NAD^+^ metabolism with NAD^+^ intermediates. Among them, nicotinamide mononucleotide (NMN), a precursor in the biosynthesis of NAD^+^, has recently received much attention from the scientific community for its anti-aging properties. In model organisms, ingestion of NMN has been shown to improve age-related diseases and probably delay death. Here, we review aspects of NMN biosynthesis and the mechanism of its absorption, as well as potential anti-aging mechanisms of NMN, including recent preclinical and clinical tests, adverse effects, limitations, and perceived challenges.

## 1. Introduction

The successful management of communicable diseases during the 20th century resulted in a significant increase in the average lifespan in several countries. The global population of people aged 65 or older is expected to increase from 10% (771 million) in 2022 to 16% (1.6 billion) by 2050. Therefore, the number of peoples aged 65 + years worldwide is projected to be more than twice the number of children under 5 years of age [1] (UN 2022). As the aging population continues to grow, age-related disorders such as hypertension, atherosclerosis, osteoarthritis, diabetes mellitus, cancer, Parkinson's and Alzheimer's diseases have become more prevalent, resulting in a substantial medical and socioeconomic burden globally [2].

There are about twelve hallmarks that contribute to the aging process and determine the phenotype of aging and longevity. These include genomic instability, telomere attrition, epigenetic alterations, loss of proteostasis, mitochondrial dysfunction, cellular senescence, altered intercellular communication, stem cell exhaustion, deregulated nutrient-sensing, disabled macroautophagy, chronic inflammation, and dysbiosis [3, 4]. Of these, genomic instability seems to be a primary cause of aging, and the accumulation of DNA damage contributes significantly to a number of disorders of early senility. The use of exogenous chemical and physical substances can negatively affect the stability and integrity of DNA, resulting in an expedited aging process [5, 6]. As we age, telomere shortens, leading to telomere attrition, and an increased risk of diseases related to the ability to renew tissues. A previous study has shown that telomerase activation can slow the progression of aging, providing insight into the close relationship between telomeres and risk of death [7]. Because epigenetic changes can affect the majority of cells and tissues, it is likely that altering or supplementing the epigenome can extend lifespan [8]. On the other hand, age-associated disorders, such as Parkinson's and Alzheimer's diseases are strongly linked to the loss of proteostasis [9]. In addition, preventing mitochondrial dysfunction may promote human lifespan extension, while cellular senescence replaces damaged tissues and potential cancer cells, showing a complex relationship between aging and lifespan extension [10, 11]. Moreover, because inflammation is associated with altered intercellular communication and stem cells play a critical role in the growth of all living organisms, thus both inflammation and stem cell depletion are major causes of aging and may prevent healthy lifespan extension [12, 13]. Macroautophagy can target not only proteins but also the entire organelles, and the decline in autophagy as a result of aging is considered to be one of the mechanisms leading to a significant reduction in organelle turnover. This justifies its discussion as a new hallmark of aging. Although all the age-related alterations mentioned above are mechanistically linked to systemic inflammation, inflammation itself is considered a hallmark of aging. Furthermore, manipulating the inflammatory and immune system in certain ways can either speed up or slow down the aging process in different parts of the body, and mutations in intestinal cells can cause dysbiosis, which disrupts the balance of the gut microbiota [4]. To manage all these features, the medical practice of age management has greatly expanded worldwide. Various medications, nutritional supplements, hormone therapies, exercise programs, and other treatments are recommended to mitigate aging and extend healthy lifespan.

NMN is an outstanding candidate synthesized from nicotinamide, an amide form of vitamin B3, and 5’-phosphoribosyl-pyrophosphate (PRPP) by nicotinamide phosphoribosyl transferase (NAMPT), showing anti-aging effects [14, 15]. NAMPT is a rate-limiting enzyme in the biosynthesis of NAD^+^, which exists in both intra- and extracellular forms, known as iNAMPT and eNAMPT, respectively [16]. A recent study reported that circulating levels of eNAMPT declined in older humans and rodents, resulting in the age-related reduction of systemic NAD^+^ levels [17]. Short-term administration of NMN has already demonstrated excellent therapeutic effects in metabolic and other diseases. NMN has been observed to attenuate damage in glucose-stimulated insulin secretion in elderly wild-type mice and certain genetic mouse models [18, 19]. In another diet-induced obese mouse model and an aging-induced type 2 diabetes model, administration of NMN potentially restores insulin action and secretion [20, 21]. In addition, NMN has been found to protect the heart against damage caused by ischemia/reperfusion injury by inhibiting the reduction of NAD^+^ that typically occurs during ischemia [22]. In aged mice, NMN maintains the progenitor/neural stem cell population and repairs arterial muscle, skeletal muscle mitochondrial function, neuronal death, and cognitive function in Alzheimer's disease [23-27]. Collectively, these evidences strongly suggest that NMN can be an effective therapeutic intervention for numerous diseases [15].

Hence, this review intends to present the biosynthesis pathways and absorption mechanism of NMN, followed by a comprehensive investigation of its therapeutic potentials with their putative mechanisms of action against aging. This will provide new insights into the possibility that NMN could be used for successful therapeutic interventions against aging in humans.

## 2. Biosynthesis and absorption of NMN

### 2.1 NMN biosynthesis

**Figure 1.**
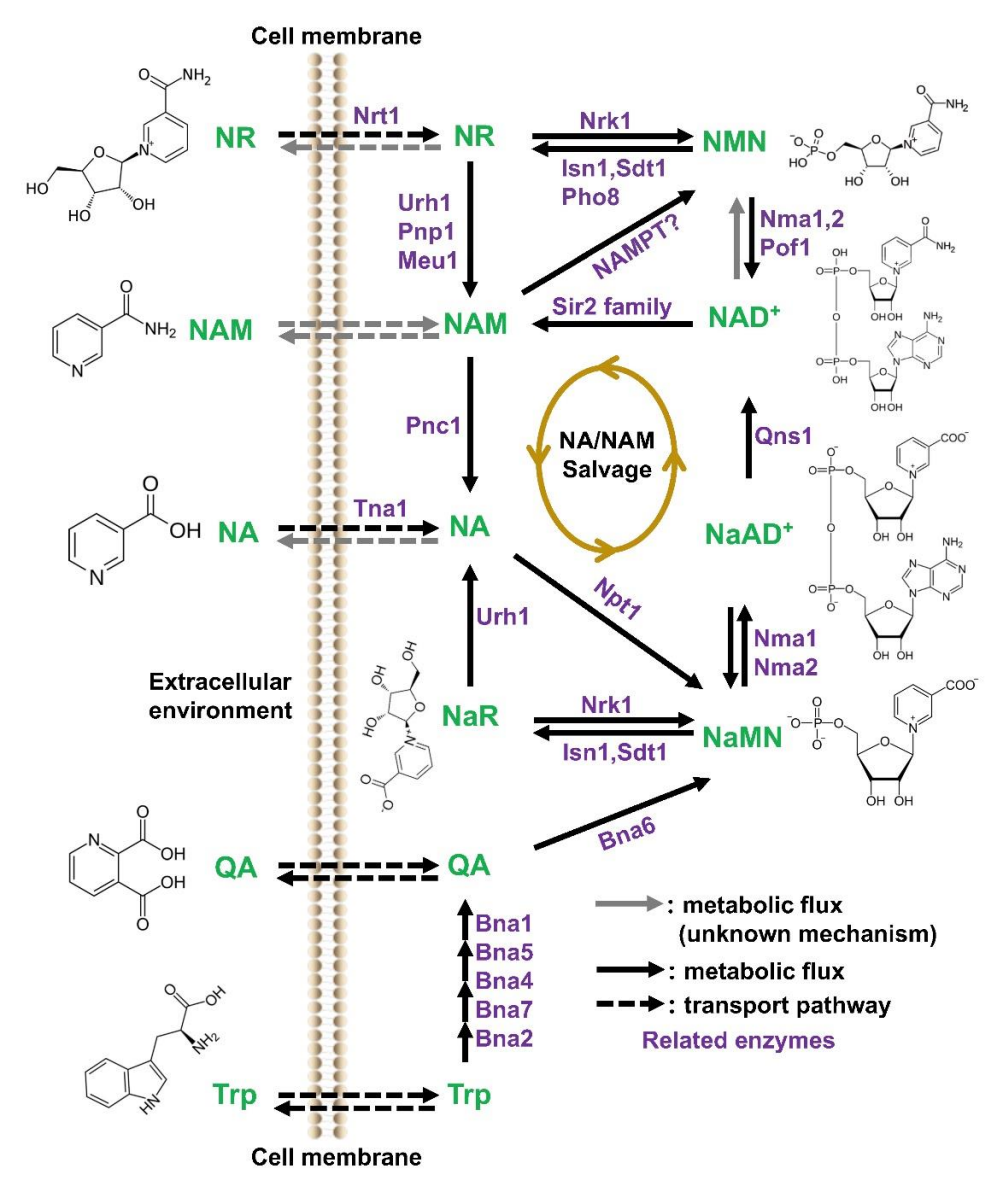
**NAD^+^ and NMN biosynthesis in yeast cells**. NAD^+^ can be synthesized by three pathways: NA/NAM salvage pathways, tryptophan de novo synthesis, and NR-mediated synthesis. Nicotinic acid biosynthesis proteins (Bna 2, 7, 4, 5, 1) are involved in the spontaneous cyclization that leads to the formation of quinolinic acid and NaMN in the tryptophan de novo synthesis pathway. However, this pathway is likely inactive when NAD^+^ levels are sufficient. In the NA/NAM salvage pathway, NaMN is produced from NA, which ultimately results in the formation of NaAD. The conversion of NaAD to NAD^+^ is facilitated by Nma1, Nma2, and Qns1. In the NR-mediated NAD^+^ biosynthesis pathway, NR is first transformed into NMN by the enzyme Nrk1. NMN is then used by the enzymes Nma1, Nma2, and Pof1 to synthesize NAD^+^. Generally, the Nrk1-dependent and Urh1/Pnp1-mediated routes assimilate NR and utilize exogenous NR made from NMN by the nucleotidase actions of Sdt1and Isn1 in the cytosol. For NMN, rate-limiting enzyme, nicotinamide phosphoribo-syltransferase, converts nicotinamide into NMN. NR kinase (NRK)-mediated phosphorylation is also used to synthesize NMN from NR. NaMN, Nicotinic acid mononucleotide; NA, Nicotinic acid; NR, Nicotinamide riboside; NaAD, Nicotinic acid adenine dinucleotide; NAD, Nicotinamide adenine dinucleotide; NMN, Nicotinamide mononucleotide; Nma, nicotinamide mononucleotide adenylyltransferase; Qns1, Glutamine (Q)-dependent NAD synthetase; NAM, Nicotinamide; Nrt1, Nitrate transporter 1; Urh1, URidine Hydrolase 1; Pnp1, Purine nucleoside phosphorylase; Pnc1, Pyrazinamidase and Nicotinamidase; NAMPT, Nicotinamide phosphoribosyltransferase; Sir2, Silent information regulator 2.

To date, different approaches have been employed for the biosynthesis of NMN. One such approach involves the chemical phosphorylation of nicotinamide riboside (NR) to NMN, which requires high levels of phosphine oxychloride and precise temperature management. In contrast, the fermentation of NMN has limitations due to its low product titers, making it unsuitable for large-scale production of NMN. Therefore, more efficient methods of NMN synthesis remain a challenging task. Since we know that NMN is a transition product of NAD^+^ biosynthesis, it is necessary to focus on NAD^+^ biosynthesis in order to fully understand the mechanisms of NMN synthesis (Fig. 1).

In mammalian cells, NAD^+^ is synthesized by the salvage pathway from nicotinic acid (NA) or nicotinamide (Nam), by the de novo pathway from tryptophan, or through the conversion of NR [28]. The degradation products of NAD^+^ such as NA and Nam are salvaged for the production of new NAD^+^ [29, 30]. The pathways involved in the biosynthesis of NAD^+^, e.g., *de novo* synthesis from tryptophan (also known as the kynurenine pathway), the salvage pathway, and the Preiss-Handler pathway. The important precursor NMN is an intermediate of the salvage-Preiss-Handler pathway involved in the biosynthesis of NAD^+^. In the salvage pathway, Nam and 5-phosphoribosyl-1-pyrophosphate are converted to NMN by NAMPT, then conjugated to adenosine triphosphate (ATP) and converted to NAD by nicotinamide mononucleotide adenylyl transferases (NMNAT) [31]. In the Preiss-Handler pathway, primarily NA is converted to NaMN through the action of enzyme nicotinic acid phosphoribosyl-transferase enzyme (NAPRT) followed by biosynthesis of NAD^+^ from NaMN utilizing NA/NaMNAT 1/2/3. Later, NAD^+^ synthetase (NADS) converts NaAD^+^ to NAD^+^ with ATP and an amino group derived from glutamine [32].

### 2.2 NMN synthesis methods

Currently, there are three main techniques used for NMN production, including chemical synthesis, fermentation method, and enzymatic catalysis. One study demonstrated that NR is converted by phosphorylation to NMN by nicotinamide riboside kinase (Nrk1 and Nrk2), which in turn can be enzymatically converted to NAD^+^ [33]. The direct conversion of NR to NMN in a single step has been considered the most favourable and effective synthetic route for NMN. In a recent study, using whole-cell Nrk-2 as a catalyst under ideal conditions, NMN was synthesized from NR with a maximum conversion rate of 98.2% [34]. This resulted in a yield of 12.6 g/L of NMN in the reaction mixture, which is considerably higher than previous synthetic methods [34]. Their study demonstrated a stable, safe, effective, and low-cost biocatalyst for NMN biosynthesis. In another study, researchers conducted an artificial *in vitro* multi-enzyme cascade bio-catalysis to synthesize NMN using starch and Nam as substrates in a one-pot process [35]. The team optimized various parameters such as buffer concentration, pH value, enzyme composition, inorganic phosphate and phosphoenolpyruvate concentration to improve the multi-enzyme cascade reaction. The authors achieved a remarkable molar yield of 87.8% for NMN with 3.2 mM Nam as a substrate.

Furthermore, an initial Nam concentration of 9.21 mM resulted in a molar yield of 55.37% for NMN [35]. These authors claim that this *in vitro* enzymatic platform offers a sustainable and environmentally friendly method for the biomanufacturing of NMN. A novel nicotinamide riboside kinase (Klm-Nrk) was identified from *Kluyveromyces marxianus*. In a recent study, researchers tried to design an environmentally friendly and low-cost biocatalytic method for converting chemically manufactured NR into NMN [36]. The team employed the novel and highly active Nrk to enzymatically synthesize NMN. A high-titer of (93.5 g/L) was achieved, using an efficient and economical ATP regeneration system, resulting in a space-time yield of 281 g/L/day [36]. The study claimed that Klm-Nrk may be an effective tool for the large-scale manufacturing of NMN [36].

The use of bacteria such as *Escherichia coli* and their lysates by a specific genotyping method is a cost-effective and simple method to produce NMN [37]. In the Gram-negative bacterium *Francisella tularensis*, NA is first transformed to NMN by NMN-synthetase, which is then adenylated to NAD^+^ by NMN adenyltransferase [38]. Another study discovered a method to effectively produce NMN in *E. coli* [39]. The authors surveyed the pathways for NMN production *in vivo*. They demonstrated that bioprospecting nicotinamide phosphoribosyltransferases (NadVs) enabled the production of 1.5 mM of NMN using the NadV from *Ralstonia solanacearum* [39]. A recent study investigated the ability of lactic acid bacteria to produce NMN either intracellularly or extracellularly [40]. The researchers utilized a bioassay that involved an auxotrophic yeast that needs NR. One of the bacterial strains had its genome sequenced, which unveiled that nicotinamide phosphoribosyltransferase, an enzyme commonly found in mammals but rarely observed in microorganisms, plays a significant role in NMN production in fructophilic bacteria [40]. In another study conducted in *E. coli*, the endogenous protein YgcS, which is mainly responsible for sugar uptake, was shown to be helpful for NMN production [41]. In this study, regulated expression of YgcS gene in an engineered strain of *E. coli* resulted in a higher production of NMN [41]. Based on the studies mentioned above, it is possible to modify some genes in the yeast model using CRIPSR technology or extract some proteins from microorganisms, especially from the genus *Rhizopus*, e.g., *Rhizopus oryzae*, to biosynthesize cheap NMN. Further details of NMN production in various models are presented in Table 1.

**Table 1:** Synthesis and obtained quantity of NMN in various models.

Model	Quantity of NMN production	References
***E. coli***	2.31 mM	[42]
***E. coli***	16.2 g/L	[43]
***E. coli***	~1.5 mM	[39]
***E. coli***	496.2 mg/L	[41]
***E. coli* (glucose and nicotinamide)**	6.79 g/L	[44]
***E. coli* (BL21(DE3) pLysS)**	17.26 mg/g	[45, 46]
***E. coli* (MG1655), Biocatalytic cascade**	1.47 mM, 2.00 mM	[47]
**Fructophilic lactic acid bacteria (auxotrophic yeast)**	2.00 mg/L	[40]
***Lactococcus lactis***	2289 μmol/L/mg	[48]
***In vitro* multienzyme cascade system**	3.0 g/L	[49]
***In vitro* multi-enzyme cascade biosystem**	3.01 mM	[35]
**Whole-cell catalysis (ATP and Mg^2+^)**	12.6 g/L	[34]

### 2.3 Mechanism of absorption of NMN

**Figure 2.**
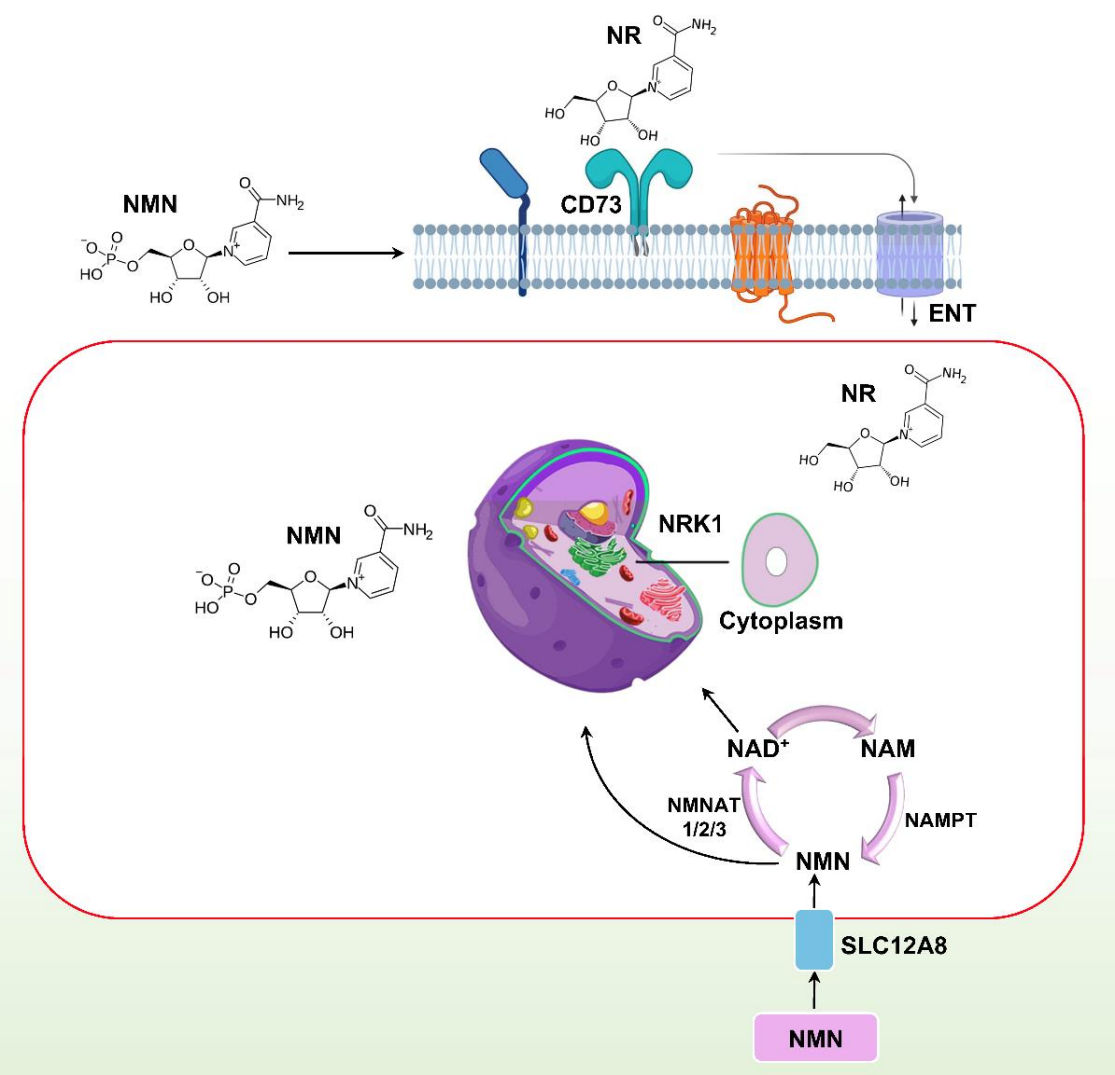
**Graphic representation of nicotinamide mononucleotide absorption in the cell**. NMN: nicotinamide mononucleotide; NR: nicotinamide riboside; Nrk 1: nicotinamide riboside kinase 1; ENT: Equilibrative nucleoside transporters; NMNAT, nicotinamide mononucleotide adenylyltransferase.

Studies have shown that, NMN needs to be converted to NR for it to cross the cell membrane and NAD^+^ synthesis [50]. NR is then converted back to NMN, which ultimately becomes NAD^+^. However, a recent discovery found a new NMN transporter, solute carrier family 12 member 8 (SLC12A8) in the gut of mice, suggesting that NMN can be directly transported into cells [51] (Fig. 2). This transporter in the lateral hypothalamus plays a crucial role in regulating energy metabolism and skeletal muscle functions, indicating its significance in the development of sarcopenia and frailty during the aging process [52]. However, further research is required to determine how much, if any, NMN passes through this transporter in humans, as there is some dispute regarding whether this transporter is actually trafficking NMN [53]. Previous studies have also shown that NMN is completely absorbed from the intestine into the bloodstream. After 15 minutes of oral administration, all NMN is absorbed into the tissues, and immediately stored as NAD^+^ in various tissues such as liver, skeletal muscle, and cortex. This ultimately induces NAD^+^ in the liver for about 30 min [54]. Furthermore, after 6 months, the authors observed spiked NAD^+^ concentration in the liver and brown adipose tissue, but not in white adipose tissue and skeletal muscle [54].

To generate NR, NMN is dephosphorylated before it enters mammalian cells. This reaction involves the extracellular receptor CD73, pyrophosphatase, and 5'-ectonucleotidase activity, which are responsible for the conversion. In cells, equilibrative nucleoside transporters facilitate the entry of NR, where it acts as an exogenous NAD^+^ precursor, as illustrated in (Fig. 2). Subsequently, the highly expressed Nrk1 facilitates the conversion of NR to NMN [50]. Based on the alterations in the NMN biosynthetic routes between prokaryotes and eukaryotes, as well as its quick absorption, NMN can be identified as one of the metabolites that plays a crucial role in NAD^+^ turnover. In general, both NMN and NR are available as supplements, but there is an ongoing debate on which one is more effective. The debate revolves around two main points: which one is more readily taken up by cells and which one is more efficiently utilized. The main difference between NMN and NR is that NMN is a larger molecule with an extra phosphate group, making it more challenging for NMN to enter the cell. However, NMN directly produces NAD^+^. On the other hand, NR more easily enters the cell, but it needs to be converted to NMN before it can produce NAD^+^.

## 3. Mechanisms involved in the anti-aging action of NMN

Aging is a progressive physiological change in an organism that leads to senescence or a decline in energy production in the mitochondria of the brain, skin, pancreas, skeletal muscle, adipose tissue, and liver in part due to a reduction in NAD^+^ levels and a failure in the organism's capability to acclimate to metabolic stress [55]. The decline in the NAD^+^ occurs due to its consumption by enzymes such as poly (ADP-ribose) polymerase (PARP), NADase (CD38/CD157), NAD^+^ dependent acetylase (sirtuins), tankyrase (TNKS), and bone marrow stromal cell antigen 1 (BST1) [56]. Among the enzymes mentioned above, CD38 is particularly important as it may play a role in the decrease of NAD^+^ associated with aging. On the other hand, sirtuins utilize NAD^+^ to perform a plethora of tasks, including deacetylase, lipoamidase, deglutarylase, desuccinylase, and demalonylase activities. Aging, lifespan extension, and age-related physiological changes are closely linked to sirtuin expression [57]. CD38 consumes NAD^+^ to form nicotinamide and ADP-ribose, while PARP utilizes NAD^+^ to make branched ADP -ribose polymers that contribute to DNA repair [14]. NAD^+^-consuming enzymes are involved in regulating various cellular activities, including mitochondrial protection, stem cell rejuvenation, and DNA repair, which are crucial for maintaining cellular health (recommended articles) [14, 58, 59]. However, these mechanisms ultimately result in a substantial depletion of NAD^+^, but the administration of NMN can balance NAD^+^ biosynthesis.

Oxidative stress and chronic inflammation usually accompany the aging process, which significantly decreases NAMPT-mediated NAD^+^ biosynthesis [21]. Phenotypic studies in laboratory animals have widely shown that NAD^+^ is replenished upon aging have been widely studied. However, the exact relationship between NAD^+^ and aging, and the mechanisms through which NAD^+^ extends lifespan, have not been fully elucidated. To investigate the interrelated molecular mechanisms of the complex functions of NAD^+^ in aging and lifespan, we can relate NAD^+^ to the twelve "hallmarks of aging" [4], and review the aging-related conditions and pathways affected by precursors of NAD^+^ in Table 2.

**Table 2:** Aging-related pathways & conditions affected by NAD^+^ precursors.

NAD^+^ precursor	Models	Aging-related pathways and conditions	References
**NR, NMN**	Worm	Activates sirtuins, which in turn deacetylate and trigger transcription factors PGC-1α, FOXOs and others, linked to aging.	[14, 60]
**NMN**	Mice	Mitigates age-associated body weight gain, improved energy metabolism, stimulated physical activity, improved insulin sensitivity and plasma lipid profile, and ameliorated eye function and other pathophysiologies.	[54]
**NR**	Worm	Restored muscle, melanocyte stem cell pools and neuronal function through initiation of the UPRmt and prohibitin protein synthesis.	[55]
**NR**	Worm, Mice, Yeast	Activate the UPRmt, causing FOXO transcription factor translocation, triggering antioxidant defenses in worms and mice, and delaying lifespan and health. In yeast model, triggering of the UPRmt has also been observed. In mice, NR treatment increased NAD^+^ levels and PGC-1α-mediated degradation of Bace1 leading to reduce Aβ production.	[19, 54, 61-63]
**NMN**	Mice	Triggered NAD^+^ and restored mitochondrial homeostasis through Sirt1-PGC-1α activation.	[24]
**NR, NMN**	Mice, Worm	NR treatment inhibited high-fat DIO by stimulating Sirt1 activity and increasing NAD^+^ levels. NMN in diabetes mouse model enhanced glucose intolerance, and increased hepatic insulin sensitivity or through restoring NAD^+^ levels. NR/NMN in a high-fat diet fed mice increased the use of lipids as substrates, improved insulin sensitivity, and increased energy expenditure.	[14, 24, 60, 64]
**NR**	HEK293 cells, Rats	Inhibits noise-persuaded hearing loss and leads to regeneration of neurite ganglia mediated by NAD^+^-dependent Sirt3 activity. Increased level of NAD^+^ directed Sirt1 activity and slows axon degeneration.	[65, 66]
**NMN**	Mice, Rho0 cells	Reverses age-associated genes expression changes linked to inflammation, partly by inducing Sirt1 activity. CD38 KO mice, NNMT KO mice and Parp1 KO mice demonstrate upregulation of NAD^+^ levels and Sirt1 activation.	[24, 67]
**NR**	Mice	Decreased senescence in neuronal and melanocyte stem cells. Enhanced mitochondrial function, dependent on Sirt1 function.	[68]
**NMN**	Human, Mice	Elongates telomere length of PBMC in mice and humans. Significantly increased the abundance of *Helicobacter*, *Mucispirillum*, and *Faecalibacterium*, and reduced *Akkermansia* richness related with nicotinamide metabolism.	[69]
**NMN, NR**	Mice, WRN-KD cells, Worm	Enhanced aging in Werner syndrome is mediated by impaired mitochondrial function and mitophagy, and that bolstering cellular NAD^+^ levels stabilize Werner syndrome phenotypes. Through DCT-1 and ULK-1-dependent mitophagy, NAD^+^ repletion helps restore NAD^+^ metabolic profiles and enhances mitochondrial quality.	[70]
**NAM**	Mice	Prevents diet-induced hepatosteatosis. Improving redox status and glucose metabolism in livers of HFD-fed mice. Reducing oxidative stress and inflammation.	[71]
**TRP**	-	Beneficial in numerous neurological disorders especially insomnia and depression. TRP supplement may exacerbate inflammation due to increased production of numerous reactive metabolites including the putative excitotoxin and N-methyl-D-aspartate (NMDA) receptor agonist quinolinic acid or QUIN.	[72, 73]
**NAM, NMN, NR, NA**	-	Preservation of cognitive function in many health contexts. Promising treatment strategy for various conditions; mainly, age-associated cognitive decline (including Alzheimer’s disease and vascular dementia), but also stroke, diabetes, and traumatic brain injury.	[74]
**NMN**	Mice, IPEC-J2 cells	Ameliorating the structural and functional deterioration in the intestine during aging. Enhanced the total antioxidant capacity, cell viability and reduced the reactive oxygen species level of senescent IPEC-J2 cells.	[75]
**NMN,NR**	BMMCs, Mice	Significantly mitigated IgE-mediated anaphylactic responses. Inhibits mast cell degranulation and anaphylaxis, however Sirt6 was required to attain these improvements.	[76]

NR, Nicotinamide riboside; NMN, Nicotinamide mononucleotide; PGC-1α, Peroxisome proliferator-activated receptor-gamma coactivator (PGC)-1alpha; FOXO, Forkhead box O; UPRmt, Mitochondrial unfolded protein response; PBMC, Peripheral blood mononuclear cell; NAM, Nicotinamide; TRP, Tryptophan; IPEC-J2, Intestinal porcine epithelial cell line J2; NA, Nicotinic acid; BMMC, Bone marrow mononuclear cells

### 3.1 NMN to NAD^+^: Pathways to anti-aging

**Figure 3.**
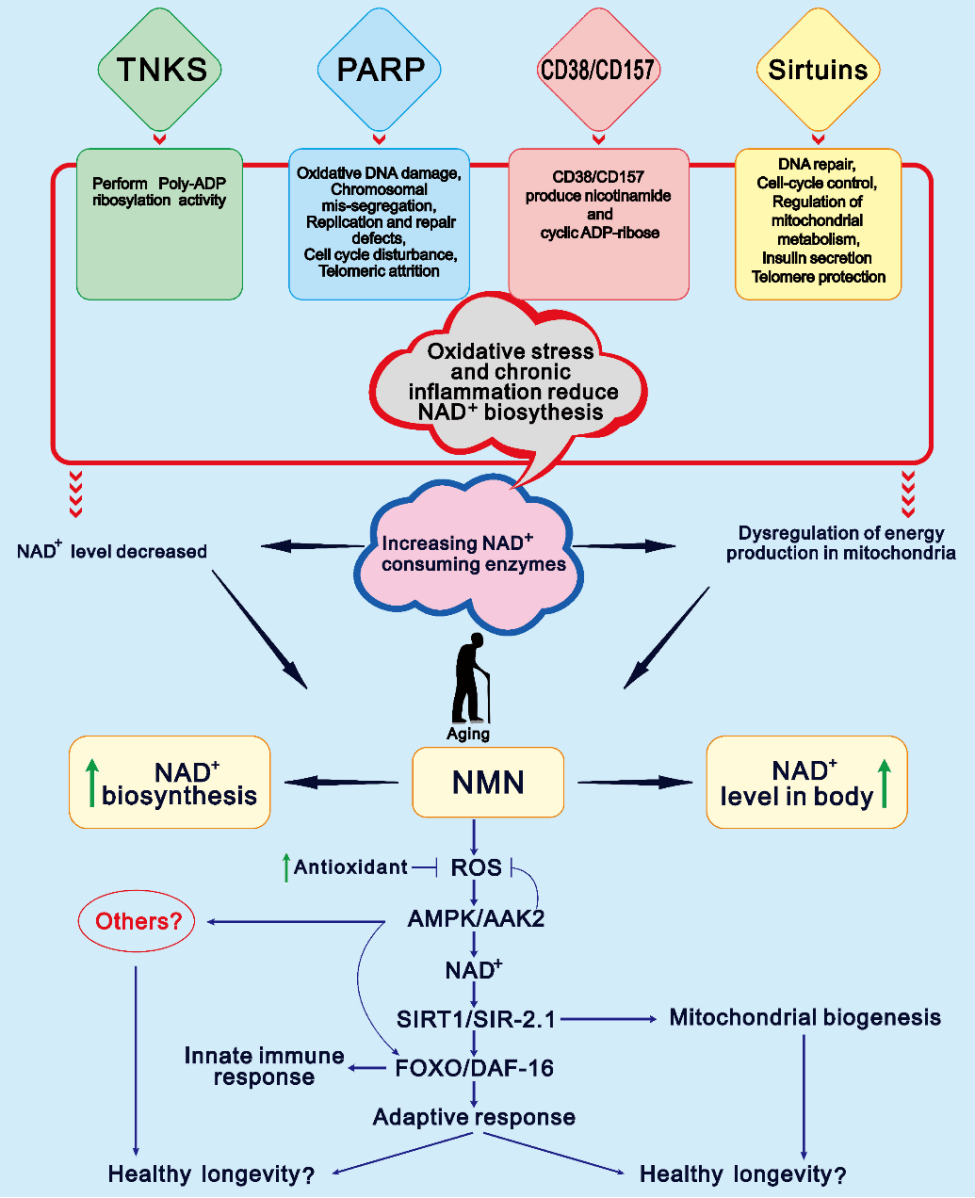
**Hypothetical model of NMN-mediated longevity and the main reasons for decreasing NAD^+^ level with age**. NMN may exploit the mechanism for lifespan extension by mitohormesis, which may depend on the AMPK/SIRT1/FOXO pathway. On the other hand, NAD^+^ degradation accelerates due to DNA damage, chronic oxidative stress, inflammation and consumption of higher NAD^+^ enzymes such as sirtuins, CD38, CD157, TNKS, poly-adenosine diphosphate ribose polymerase, etc. When NAD^+^ levels decrease, dysregulation of mitochondrial energy production may occur, leading to aging and decreasing lifespan extension. Supplementation with NMN reverses the aging process, promotes NAD^+^ levels, and prevents age-related disorders.

Aging causes biological changes such as cognitive decline, inhibition of the sirtuin gene, and DNA damage, all of which can be prevented by increasing the level of NAD^+^ in the organisms’ body [24]. NAD^+^ levels may increase in the body even in the absence of NMN supplementation, in response to conditions associated with lower energy intake [77], fasting, calorie restriction, low blood sugar levels, and exercise. Although, ingestion of a high-fat diet (HFD) and aging cause a decrease in the level of NAD^+^ [68]. Previous studies have shown that NMN can induce Sirt1 activity and can be used as a dietary supplement [78]. On the other hand, when NAD^+^ levels are low, Sirt1 is incapable of preventing the activation of hypoxia-inducible factor 1 alpha (HIF-1), and higher HIF-1 levels block nuclear-mitochondria contact at the cellular level and at the systemic level between the hypothalamus and adipose tissue [79]. In this case, the disruption of mitochondria-nucleus communication can lead to a decline in mitochondrial function, resulting in age-related abnormalities and disease. However, mitochondrial function and especially mitochondrial-nuclear communication can be stimulated by the administration of NMN [24]. According to a previous study, the aging process is characterized by a reduction in the production of NAD^+^ and an increase in its consumption, impairing functions of different tissues [80]. The authors also showed that NMN can enhance NAD^+^ metabolism and has the potential to ameliorate age-related pathological conditions *in vivo*. In another study, NMN supplementation was shown to restore Sirt1and NAD^+^ levels, ultimately protecting kidneys from age-related acute kidney injury (AKI) in a Sirt1-dependent manner [81]. Instead, mitohormesis, and particularly a mechanism dependent on the AMPK/SIRT1/FOXO pathway is important in the aging process. Researchers have also discovered a connection between NAD, sirtuins, and FOXO that increases lifespan in *Caenorhabditis elegans* [82]. Therefore, we propose a hypothetical mechanism that NMN might extend lifespan via AMPK/SIRT1/FOXO signaling and mitohormesis, as well as the reasons for the decrease in NAD^+^ levels during aging (Fig. 3).

### 3.2 Effects of NMN on DNA repair

DNA repair is important for cell survival, vitality, and disease prevention. However, the ability of cells to restore damaged DNA decreases as one ages, and the underlying reasons for this decline are still not completely understood. Recent research has shed light on how and why the body's ability to repair DNA declines over time, suggesting an unspecified role for NAD^+^ as a critical regulator of protein-to-protein interfaces in DNA repair. A previous study showed that treatment with NMN is linked with telomeres length and decreased DNA damage response [83]. The authors demonstrated that administration of NMN sustained the length of telomere, reduced the DNA damage response and p53, and improved mitochondrial function in a manner that partially depended on Sirt1 in mice [83]. Moreover, DNA damage occurs in various types of internal injury, and targeting this damage could be an efficient approach to reducing aging. A recent study has shown that NMN supplementation could potentially reduce hydrogen peroxide- and hypoxia-induced tubule cell DNA damage and subsequent cellular senescence in human proximal tubule cells [84]. They also confirmed the inhibition of DNA damage, anti-aging effect, and anti-inflammatory effect of NMN in a mouse model of unilateral ischemia-reperfusion injury (uIRI) [84]. Recently, it was found that NMN could substantially decrease nude mice body weight and induce repair of DNA during lung cancer, however, NMN in a mouse xenograft model did not restrain tumor growth, nor did it promote tumor growth [85]. In addition, as we know that NAD^+^ binds to the Nudix homology domain of numerous proteins, but its exact role is not yet clear. A previous study showed that Deleted Breast Cancer protein1 (DBC1) has this domain, which restricts the activity of PARP, a known DNA repair protein [86]. NAD^+^ interacts with the nudix domain of DBC1, hence reversing PARP1 inhibition. However, during aging when the levels of NAD^+^ decreases the ability of PARP1 to repair DNA declines. This mechanism was studied in mice with DNA damage caused by gamma radiation. The old and young mice were administered 500 mg/kg/day NMN intraperitoneally. The study showed that NMN improved the hepatic NAD^+^ concentration as well as the activity of PARP1 and repaired DNA damage [87]. These findings suggest that NMN could enhance the bioavailability of NAD^+^ and specifically promote DNA repair.

### 3.3 Mitochondrial health and NMN

Mitochondrial quality and abundance are critical for health, healthy lifespan, and longevity, and its dysfunction is considered a hallmark of aging, manifested in various age-related conditions [59]. NAD^+^ and NAD^+^-mediated deacetylase activity have been identified as positive regulators of mitochondrial function. On the other hand, mitochondrial dysfunction is commonly observed in cases of heart failure, which highlights the significance of enhancing or repairing mitochondrial health as a potential therapeutic strategy. A previous study used a heart-specific mouse model lacking Krüppel-like factor 4 (KLF4) that was sensitive to stress. The study found that, prior to stress, hyperacetylation of mitochondrial proteins was related with decreased NAD^+^ and Sirt3 in the heart, demonstrating that the KLF4-deficient heart is causally associated with NAD^+^-related abnormalities [88]. At the mechanistic level, the authors demonstrated that administration of NMN preserved the ultrastructure of mitochondria, decreased ROS levels, and stopped cell death in the heart [88]. In another study, researchers used a combination of two different mitochondria-targeting drugs, SS-31 and NMN, and found that SS-31 moderately reversed age-associated diastolic dysfunction, while NMN completely reversed age-related insufficiency of systolic function at higher workloads [89]. They also pointed out that both SS-31 and NMN are effective in restoring various features of cardiac and mitochondrial health. The combination of both drugs resulted in a synergistic effect that best restored old hearts to the young state [89].

Furthermore, studies have shown that ovarian aging can impact the reproductive health of older women, leading to a decline in egg quality that is associated with mitochondrial dysfunction. In a recent study, long-term administration of NMN to 4, 8, 12, 24, and 40-week-old female ICR mice showed anti-inflammatory and anti-aging effects on organ structures, as well as improvement in endocrine function and estrous cycle status [90]. This study also showed that autophagy level, mitochondrial biogenesis, and protease activity in granulosa cells improved after 20 weeks of NMN administration [90]. In another study, long-term NMN administration efficiently attenuated the age-related physiological decline in C57BL/6N mice [54]. The researchers also found that NMN had the ability to reverse age-related alterations and gene expression in major metabolic organs, as well as enhance mitochondrial oxidative metabolism and restore the balance of mitonuclear protein levels in skeletal muscle [54]. Recent studies have also shown that Leigh syndrome, a mitochondrial disorder, can be treated with NMN supplementation in *Ndufs4*-KO mice, a mouse model of Leigh syndrome [91]. In addition, the study found that NMN preserves mitochondrial function and increases survival during hemorrhagic shock in a rodent model [92]. Hemorrhagic shock leads to a decrease in the level of NAD, causing metabolic imbalance. However, the administration of NMN significantly attenuates inflammation, promotes cellular metabolism, and supports rodents’ survival after hemorrhagic shock [92]. In addition, a study showed that NMN alone or in combination with melatonin, can improve mitochondrial function and reverse age-related memory and learning deficits in the brains of aged animals [93]. These findings collectively suggest that NMN can increase the bioavailability of NAD^+^ and restore mitochondrial health, thereby inhibiting one of the hallmarks of aging "mitochondrial dysfunction".

### 3.4 NMN and autophagy

Autophagy plays a key role in aging and longevity. The main role of autophagy in aging and health includes cellular energy, cellular homeostasis, neuroprotection, and anti-inflammatory responses [94, 95]. Indeed, impaired autophagy is the major sign of aging that may contribute to age-related disorders [59, 94, 95]. It has been shown that the upregulation of autophagy can limit the development of several age-related disorders in animals, such as Parkinson's and Alzheimer's diseases [95-97]. Likewise, autophagy inhibitors have been found to shorten the lifespan of *Drosophila*, yeast, and nematodes due to changes in proteins involved in the autophagy pathway, such as Atg1 (*unc-1* in *C. elegans*) [98, 99]. This relationship between autophagy induction, aging, and cell death is evident in various diseases, such as cancer, neurodegeneration, inflammatory diseases, and infections.

NAD^+^ and its precursors, especially NMN, are crucial for the induction of autophagy and may act as an important regulator in the underlying molecular mechanism. In a recent study, NMN and NR were shown to increase the level of NAD^+^, which in turn restored mitophagy to normal levels in worms with Werner syndrome [70]. The authors demonstrated that decreased mitophagy accelerates the occurrence of impaired mitochondria in Werner syndrome because mitophagy eliminates damaged mitochondria. In this case, treatment with NR and NMN increases longevity, mitigates aging, and improves mitophagy in a Werner syndrome model organism and human cells [70]. Mitophagy is essential for healthy longevity and mitochondrial homeostasis [100]. The induction of mitophagy via NMN/NR was also reported in 2014 [101], and has since been observed both *in vivo* and *in vitro* in different models [102-104]. A comprehensive summary of how NMN/NR triggers mitophagy/autophagy, including transcriptional, translational, and posttranslational regulation of genes and proteins associated with the mitophagy/autophagy process, is available elsewhere [105]. As mentioned earlier, NMN can enhance DNA repair, thus NMN likely helps to connect mitochondrial maintenance and nuclear DNA repair [59]. Similarly, NMN may induce mitophagy/autophagy, which may help delay aging and prolong healthy life expectancy in certain models.

## 4. NMN in aging and health improvement: the leading benefits

### 4.1 Mitigate aging

Although NMN was discovered in 1963, interest in research on NMN as an anti-aging agent did not emerge until about ten years ago. NMN research in laboratory animals gained widespread notoriety in the early 2011 when a study demonstrated NMN's ability to improve blood sugar control [21]. As we know, NAD^+^ is considered comparatively safe and has shown excellent abilities in treating and preventing various diseases, including aging. NMN has shown its potential to improve various functions, such as kidney, liver, heart, skeletal muscle, and nerve function, as well as showing potentially effective results in inflammation and immunity [14, 15, 106]. In rodent model, many studies revealed that administration of NMN increases the biosynthesis of NAD^+^ in numerous tissues, such as liver [107], pancreas [21], heart [108], kidneys [81], eyes [109], testis [110], skeletal muscles [24], and blood vessel [23]. Furthermore, studies have shown that NMN slows aging by increasing NAD^+^ levels in a variety of ways [111], and mechanisms that help to delay aging and restore organelle functions [62].

### 4.2 Support cognitive functions

The administration of NMN can increase NAD^+^ levels in the hippocampus and hypothalamus within 15 min, suggesting that NMN may also cross the blood-brain barrier (BBB) to synthesize NAD^+^ in the brain [112, 113]. A study has shown that NMN inhibits the buildup of beta-amyloid plaques, which are the symptoms of neurological disorders [27]. Another study demonstrated that older mice supplemented with NMN had better spatial working memory, better neurovascular health, better gait coordination, and improvements in other essential components of brain function [111]. However, the effect of NMN on the brain and whether it can surely cross the BBB, as well as the possible mechanisms, need further investigation to provide clarity.

### 4.3 Support reproductive health

In mammals, aging of the female genitalia is characterized by a marked decline in the quantity and quality of follicles and oocytes. Poor-quality oocytes are a common problem in older women, leading to poor reproductive outcomes. Researchers have shown that long-term NMN supplementation significantly improved the morphology and number of oocytes in aged ovaries, which confirmed that NMN could partially restore and enhance the reproductive capability of animals [114]. In another study, HFD showed adverse effects on plasma glucose removal in the postpartum and postweaning period, with glucose tolerance of mice supplemented with HFD after weaning significantly improved by NMN injection [115]. If these results are replicated in humans, it would indicate that supplemental NMN could help women have pregnancies with healthy fetuses at advanced ages. In addition, supplementation with low doses of NMN could potentially be a safe and non-invasive method to enhance fertility and aid in maintaining healthy pregnancies as women get older. Although these findings are promising, human clinical trials will be needed to determine whether NMN improves fertility in middle age.

### 4.4 Improve metabolic markers and healthy weight management

NMN has been shown to increase Sirt3 activity, hepatic mitochondrial lipid oxidation in circadian mutant mice, and hepatic citrate synthase function in obese HFD mice [107, 116]. A critical aspect of these results is that older animals seem to respond very well to NMN treatment. This is because NMN supplementation improves HFD-induced glucose intolerance and dyslipidemia, endothelial function, and mitochondrial oxidative metabolism in the skeletal muscle [21, 23, 24]. A recent study in humans shows that NMN supplementation improves numerous markers of glucose metabolism in skeletal muscle that are often impaired in metabolic disorders in humans [117]. Previous animal studies have also demonstrated the metabolic health benefits of NMN. Mice supplemented with NMN for 12 months showed significantly improved insulin sensitivity. Another study reported that the addition of NMN to mice with diabetes led to notable improvement in their levels of NAD^+^, insulin sensitivity, and glucose tolerance [54]. Hence, it would be very interesting to compare the metabolic effects of NMN in younger mice with those in older mice.

## 5. Clinical studies of NMN related to aging and health improvement

### 5.1 Clinical studies related to the safety and bioavailability of NMN

Recently, several clinical trials have evaluated the safety and bioavailability of NMN. A recent, non-blinded clinical trial assessed the NMN safety in 10 healthy men who were administered with 100, 250, and 500 mg of NMN orally, and their clinical parameters were assessed [118]. The authors note that each NMN dose was successfully metabolized without adverse effects or fluctuations in heart rate, blood pressure, body temperature and oxygen saturation [118]. In another placebo-controlled, randomized, double-blind, parallel-group study, the authors investigated the efficacy of increasing NAD^+^ and the safety of orally administered NMN in 30 healthy subjects. The healthy subjects were supplemented with 250 mg/day of NMN for 12 weeks, and physiological and laboratory tests were performed [119]. Orally administered NMN didn't cause any abnormalities, and the laboratory tests of all subjects were found to be normal. The authors also found a significant rise in the whole blood NAD^+^ levels of all subjects and suggested NMN as a safe strategy to increase NAD^+^ levels in humans [119].

Further human studies are required to determine the safety of intravenous introduction of NMN into the body. In particular, sublingual administration of NMN could avoid first-pass metabolism and thus protect NMN degradation. However, no human or mouse studies have been conducted to support this route of administration. Further research will help determine if this is the best and most effective route for NMN delivery. Bioavailability is a key efficacy indicator that shows how blood NAD^+^ levels change after NMN supplementation. The bioavailability of NMN is currently being investigated in human volunteers [119].

### 5.2 Clinical studies of NMN related to aging-conditions

Following safety and bioavailability studies in human volunteers, numerous clinical trials have been conducted to explore the efficacy of NMN in aging. A recent double-blind, parallel, randomized controlled trial was conducted on 66 healthy volunteers aged between 40 and 65 years [120]. The healthy volunteers were instructed to take two capsules (comprising 150 mg NMN/day) for 60 days. The study found that serum levels of NAD^+^/NADH increased by 11.3% on day 30, compared to the placebo group [120]. On day 60, the author found that the NAD^+^/NADH level increased by an additional 38% compared to baseline, which increased by only 14.3% in the placebo group. The homeostatic model for insulin resistance (HOMA IR index) also showed an increase of 0.6% in the NMN group, and 30.6% in the placebo group from baseline [120]. Finally, the study's findings suggest that NMN has the potential to boost NAD^+^ levels in cells and improve insulin sensitivity, as indicated by the increase in NAD^+^/NADH levels observed at days 30 and 60, which in turn may lead to higher energy levels and anti-aging effects [120].

Mental and physical fatigue, as well as poor sleep quality in older adults have been linked to a shorter life expectancy and more deaths. In a recent randomized, double-blind, placebo-controlled trial, 108 participants were supplemented with NMN (250 mg) or a placebo once daily for 12 weeks. Their sleep quality, fatigue, grip strength, and physical performance were assessed [121]. The study found that NMN intake efficiently enhanced the function of lower limb and decreased sleepiness in adults. These findings suggest that NMN may help prevent declines in physical performance and fatigue in adults [121].

To date, one clinical trial of NMN in hypertensive patients is ongoing at the First Affiliated Hospital of Sun Yat-sen University in China (NCT04903210), which is in the recruitment phase, but the results have not yet been published. In addition, a recent study evaluated the efficacy of NMN in promoting metabolic benefits in overweight or obese individuals. In this study, 13 obese or overweight postmenopausal women with a history of prediabetes received 250 mg of NMN orally daily for 10 weeks, while 12 other women received a placebo daily for the same period [117]. The researchers showed that NMN supplementation of 250 mg/day increased insulin signaling, muscle insulin sensitivity, upregulated platelet-derived growth factor receptor β expression, and some other genes associated with muscle remodeling. Their positive results have opened the door for further research to potentially gain metabolic benefits and may be treat diabetic patients with NMN [117].

## 6. Possible adverse effects of NMN

Although the advantages of NMN are apparent, certain studies suggest that individuals should exercise caution when using it. NMN is generally well-tolerated at low doses, but high doses may have adverse effects. In mice, long-term administration of NMN in water for 12 months showed no deleterious effects [54]. In another study, 500 mg of NMN in a single oral dose was found to be safe and well-tolerated in healthy Japanese men, with no significant adverse effects [118]. However, some concerns about the study design, such as the absence of a placebo group and the testing of only one dose, strongly suggest the need for further clinical trials with NMN. In these trials, both therapeutic and toxic dose ranges should be carefully identified, and both men and women and healthy and sick subjects should be included [118]. Possible adverse effects of NMN have been warned, particularly when administered at high doses, including cancer growth and hepatic pressure, as reviewed by Reiten and colleagues [122]. Previous study revealed that an overdose or high dose of NMN may have an effect on some aspects of fertility [123]. Furthermore, administration of NMN through intraperitoneal injection resulted in elevated levels of oxidative stress in the sperm, and a decline in sperm quality among male offspring of obese mothers who were subjected to an HFD. However, these effects were not observed with oral administration of NMN [124]. This indicates that the effects of NMN are complex, and that further studies are needed to determine the appropriate mode of administration and therapeutic dosage in the future.

Although data from preclinical studies have propelled the field toward human studies, the safety profile, pharmacology, and NMN efficacy are currently under evaluation in clinical trials [122]. The easy availability of these molecules to the public and their current use by lots of people raises a key question that without long-term data from clinical trials and comprehensive investigation, could NMN itself or other NAD^+^ precursors that elevate NMN levels potentially harm individuals, especially given that NMN has been shown to be toxic under certain conditions?

## 7. NMN and human health: the landscape of the future

**Figure 4.**
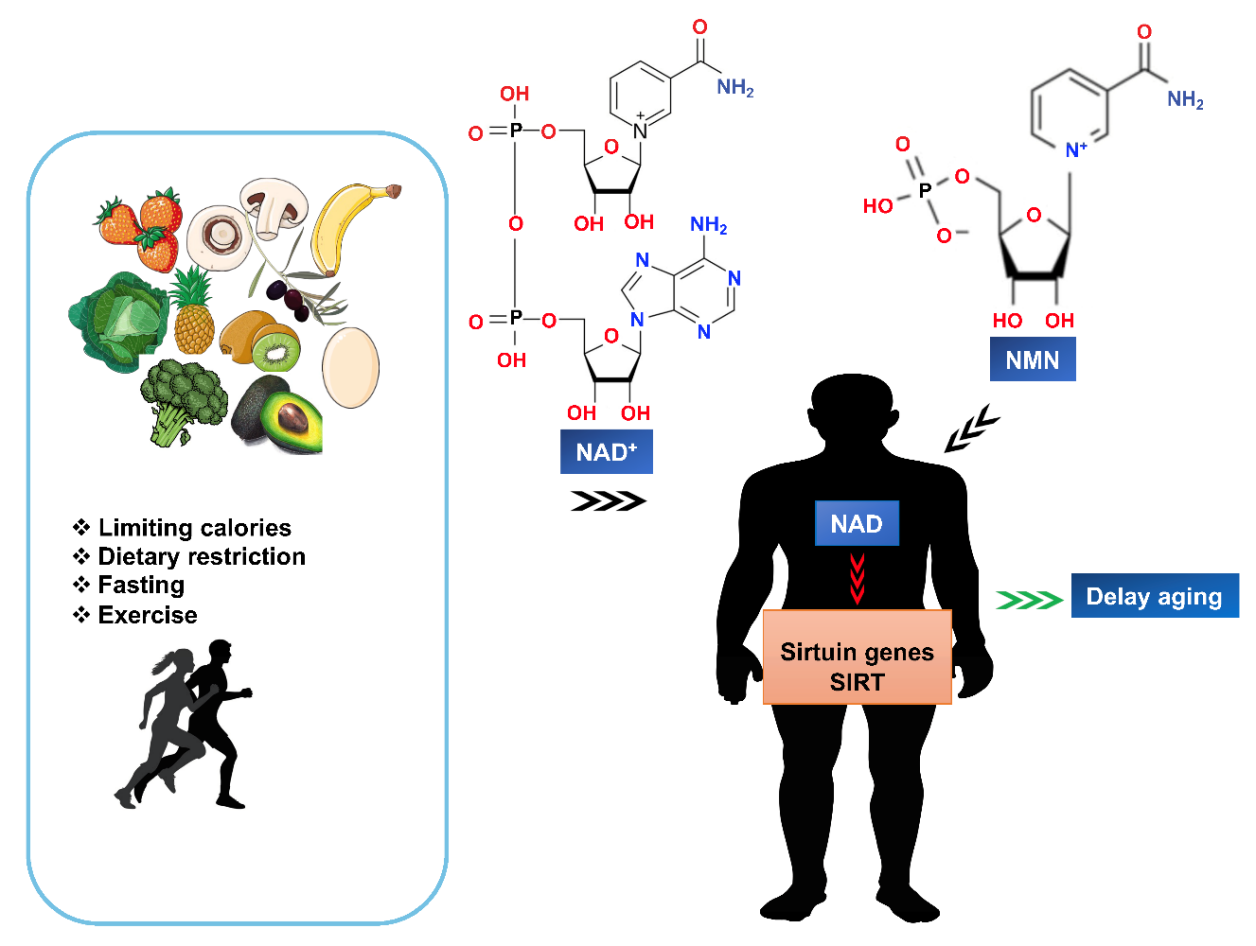
NMN increases NAD^+^, activates sirtuins, leading to delaying of aging.

Mice are not humans, but the results of a mouse study published in 2018 gave researchers encouragement for increasing human lifespan. In that study, the authors reported that NMN significantly restored faltering blood flow in aging mice, which is a major component of aging [125]. Initially, NMN improved blood flow similar to exercise, by stimulating a group of molecules called sirtuins [125]. Furthermore, NMN supplementation increased Sirt1 activity, which in turn promoted mitochondrial health, and ultimately led to increased cellular energy production [58]. NAD^+^ activates sirtuin, which are present in the cells of all organs and tissues of living beings and is used for energy production (Fig. 4). NAD^+^ was first mentioned in the literature in 1906 as a substance that facilitates the fermentation of yeast alcohol. Several types of sirtuins control aging in humans, but they are usually latent and inactive and require activation or stimulation. Simply put, the signs of aging are a deterioration in tissue or organ function, usually caused in part by a decrease in NAD^+^. On the other hand, NAD^+^ levels can be stimulated by exercise or fasting, but the intake of NAD^+^ itself does not allow the transport of intact NAD to the cells. Therefore, researchers discovered that "NMN", which occurs naturally in breast milk and is the first nutrient that humans ingest, can help restore human body functions. It is known to be converted to NAD^+^ in the body when consumed. In the past, NMN has been shown to have a significant anti-aging effect and showed promising results in animal studies related to heart failure, kidney failure, Alzheimer's disease, diabetes, and more. Since all of these results are from animal studies, it may be too early to assume a "rejuvenating" effect in humans.

In this context, some interesting questions are to be addressed in the future, such as what are the specific molecular mechanisms by which NMN acts on mitochondrial homeostasis? NMN/NAD^+^ activates the mitochondrial unfolded protein response, which is anti-aging/anti-Alzheimer’s disease under many conditions and can lead to increased mitophagy [126], but the underlying molecular mechanisms still need to be further elucidated. In addition, the intracellular transporters for NMN need to be better understood. What is the exact therapeutic dose of NMN required for various diseases in clinical trials? Is there a risk associated with long-term NMN supplementation in humans? If NMN shows clinical benefit, what further clinical trials or combinations with other drugs should be conducted to mitigate aging?

### 7.1. Limitations and challenges in NMN research

Research on the use of NMN as a therapeutic drug for aging and lifespan extension has been increasing, and various researchers have made great efforts to fully understand the anti-aging mechanisms of NMN. However, despite these efforts, there are still various limitations and challenges that exist. The development and delivery of products containing NMN encounter various obstacles and difficulties. At room temperature, NMN is normally stable in water and is readily absorbed when administered to mice via oral gavage, resulting in a rapid rise of NMN in the plasma [127]. However, there are variations in NMN metabolism across different tissues [128], and studies suggested that the gut microbiome may delay the gastrointestinal uptake of NMN when administered orally [129, 130]. Therefore, it is important to conduct additional research using high-throughput techniques to investigate the regulation of NAD^+^ balance and the impact of NAD^+^ and its precursors, especially NMN, on the epigenome, proteome, metabolome, and transcriptome. Moreover, establishing the therapeutic and toxic dose ranges of NMN is a difficult task, so further clinical studies are needed to rigorously establish these ranges. On the other hand, many NMN products are available in the market, but their cost is very high, making them difficult to afford for low-income populations and countries. Hence, there is an urgent need for low-cost production methods for NMN. In addition, several potential drugs have been discovered through fundamental and translational research that could target aging processes. Some of these drugs are currently being used clinically for other purposes. However, designing clinical trials to test interventions aimed at aging processes presents a distinct set of challenges. Hence, researchers need to develop concepts for conducting clinical trials of aging interventions such as NMN. Additionally, researchers should present strategies for translating biological discoveries into treatments that can prevent, delay, mitigate, or treat age-associated diseases.

## 8. Conclusions and future outlooks

In recent years, the growing number of elderly people has put tremendous socioeconomic pressure on many countries, societies, families, and healthcare systems. On the other hand, changes in diet, dietary restriction [131], exercise, and fasting are examples of lifestyle changes that can improve health and increase the life expectancy of individuals. Despite these benefits, very few people follow these steps due to various lifestyle difficulties. Thus, the outstanding effects of NMN addressed in this paper could promote healthy aging and delay the onset of many age-related diseases. We also propose that NMN is potentially effective in ameliorating age-related disorders due to its recognized DNA repair, mitochondrial health, autophagy induction, and several other potential properties. In addition, NMN supports cognitive function and reproductive health, as well as improves metabolic markers and healthy weight management. These properties encourage that NMN could be used as a successful therapeutic anti-aging intervention in humans.

On the other hand, various NMN-related health products are widely available in the market. However, the prices of these products are extremely expensive, and efficient and simple methods for NMN synthesis or purification are limited. Therefore, low-cost production methods for NMN are urgently needed.

NMN has shown great potential as a molecule that can enhance NAD^+^ metabolism and mitigate age-associated pathological conditions *in vivo*. This potential has driven NMN to the clinical trial stage. Therefore, further research into the NAD^+^ metabolism pathway and the applications of NMN is fascinating and deserves further investigation. In addition, the accurate dosage, pharmacology, toxicology, and safety profile of NMN in humans should be investigated as a priority. While many studies on NMNs related to aging are ongoing, several interesting questions remain to be addressed. In the future, researchers should develop more healthy or anti-aging products, and can make use of existing medications and improve current production methods. After administering NMN, future studies should focus on known targets and explore novel targets for drug development. Additionally, researchers should investigate the effects of combining NMN with other precursors or extracts to determine their impact on key outcomes such as DNA repair, mitochondrial health, and autophagy. With solid research, NMN supplementation could be a potential multi-functional strategy for an aging population.
